# Development of the SF-6Dv2 health utility survey: comprehensibility and patient preference

**DOI:** 10.1186/s41687-022-00455-w

**Published:** 2022-05-12

**Authors:** Lynne Broderick, Jakob B. Bjorner, Miranda Lauher-Charest, Michelle K. White, Mark Kosinski, Brendan Mulhern, John Brazier

**Affiliations:** 1 QualityMetric, 1301 Atwood Avenue, Johnston, RI 02919 USA; 2 University of Copenhagen, Copenhagen, Denmark; 3 Centre for Health Economics Research and Evaluation, University of Technology Sydney, Sydney, Australia; 4 School of Health and Related Research (ScHARR), University of Sheffield, Sheffield, UK

**Keywords:** Patient-centered research, SF-6D, SF-6Dv2, Utility score, SF-36v2, Preference-based measure

## Abstract

**Background:**

The SF-6Dv2 classification system assesses health states in six domains—physical functioning, role function, bodily pain, vitality, social functioning, and mental health. Scores have previously been derived from the SF-36v2® Health Survey. We aimed to develop a six-item stand-alone SF-6Dv2 Health Utility Survey (SF-6Dv2 HUS) and evaluate its comprehensibility.

**Methods:**

Two forms of a stand-alone SF-6Dv2 HUS were developed for evaluation. Form A had 6 questions with 5–6 response choices, while Form B used 6 headings and 5–6 statements describing the health levels within each domain. The two forms were evaluated by 40 participants, recruited from the general population. Participants were randomized to debrief one form of the stand-alone SF-6Dv2 HUS during a 75-min interview, using think-aloud techniques followed by an interviewer-led detailed review. Participants then reviewed the other form of SF-6Dv2 and determined which they preferred. Any issues or confusion with items was recorded, as was as overall preference. Data were analyzed using Microsoft Excel and NVivo Software (v12).

**Results:**

Participants were able to easily complete both forms. Participant feedback supported the comprehensibility of the SF-6Dv2 HUS. When comparing forms, 25/40 participants preferred Form A, finding it clearer and easier to answer when presented in question/response format. The numbered questions and underlining of key words in Form A fostered quick and easy comprehension and completion of the survey. However, despite an overall preference for Form A, almost half of participants (n = 19) preferred the physical functioning item in Form B, with more descriptive response choices.

**Conclusion:**

The results support using Form A, with modifications to the physical functioning item, as the stand-alone SF-6Dv2 HUS. The stand-alone SF-6Dv2 HUS is brief, easy to administer, and comprehensible to the general population.

## Introduction

Health-related quality of life (HRQoL) is a multi-faceted concept specifically related to how one’s health affects overall quality of life as it pertains to their physical, mental, emotional, and social functioning [1]. Measures of HRQoL can be categorized into 2 types: health profile measures and preference-based health utility measures [2]. Profile measures provide scores for each domain of health that is measured. Examples of heath profile measures include the SF-36 Health Survey (SF-36) [3] and the SF-36v2® Health Survey (SF-36v2) [4]. Alternatively, health utility measures summarize ratings of multiple health domains into a single preference-based score anchored by the values 0 and 1, where 0 = death and 1 = perfect health [5]. These preference-based health utility measures have become increasingly valuable for calculating quality adjusted life years (QALYs), and are widely used in clinical trials and in determining the value and benefits of health care.

The SF-6D is one of the most widely used preference-based health utility measures [6], along with the EuroQol-5D (EQ-5D) [7], and the Health Utilities Index (HUI) [8, 9]. Each of these measures is unique in terms of the domains measured, the items, and the preference weights used to determine scoring. The scoring algorithm of the SF-6D is based on studies assessing the value individuals place on different health limitations. Such studies use hypothetical scenarios where individuals trade between different health states [6, 10, 11]. The SF-6D is based on 6 health domains: physical functioning, role functioning, bodily pain, vitality, social functioning, and mental health. Since its development, researchers have validated county-specific value sets of the SF-6D for populations in the United Kingdom, Brazil, China, Japan, and Portugal [12–16]. Additionally, improvements have been made to the scoring algorithms for the SF-6D resulting in the development of the SF-6Dv2 [12, 17]. The SF-6Dv2 score is derived from 10 items in the SF-36v2. Compared to the SF-6D, the SF-6Dv2 describes more distinct levels of health, reduces floor effects, and provides clearer wording for health state valuation scores [12, 17].

The updated scoring algorithm of the SF-6Dv2 highlighted the need for a stand-alone measure with reduced respondent burden. A stand-alone SF-6Dv2 health utility measure eliminates the need to administer all 36 items in the SF-36v2 in order derive an SF-6Dv2 score. To address this need, 2 stand-alone forms of the SF-6Dv2 Health Utility Survey (SF-6Dv2 HUS) were developed: Form A and Form B. During the initial development of the standalone measure, we wanted to test whether respondents preferred a measure that aligns with the question type format of the SF-36v2 or a measure that resembles other health utility measures (e.g., the EQ-5D). We opted to create and subsequently test two versions of the SF-6Dv2, to learn which presentation is easiest to understand and complete by respondents. Form A asks users to answer 6 questions (one per health dimension, by selecting from 5 to 6 response choices each; similar to the formatting of the SF-36v2); Form B relies on headings to identify each of the 6 health dimensions and asks users to review 5–6 descriptive statements for each health domain and select the one that best describes them (see Table 1).

**Table 1:** Overview of SF-6Dv2 Forms A and B

Category	Form A	Form B
Instructions	The next six questions ask about different aspects of your health. For each question, please select the one response that best describes your health	The next six items concern different aspects of your health. For each item, please select the one statement that best describes your health
Physical functioning	1. *Does your health now limit you in your physical activities*, for example vigorous activities (such as running, lifting heavy objects, participating in strenuous sports), moderate activities (such as moving a table, pushing a vacuum cleaner, bowling or playing golf), or bathing and dressing? Not limited at all in vigorous activities Limited a little in vigorous activities Limited a little in moderate activities Limited a lot in moderate activities Limited a lot in bathing and dressing	*Physical functioning* Your health does not limit you in vigorous activities (such as running, lifting heavy objects, participating in strenuous sports) Your health limits you a little in vigorous activities Your health limits you a little in moderate activities (such as moving a table, pushing a vacuum cleaner, bowling, or playing golf) Your health limits you a lot in moderate activities Your health limits you a lot in bathing and dressing
Role functioning	2.* During the past 4 weeks, how much of the time have you accomplished less than you would like at work or during other regular daily activities as a result of your physical health or emotional problems?* None of the time A little of the time Some of the time Most of the time All of the time	*Role functioning (Ability to work or do regular daily activities) in the past 4 weeks* You accomplished less than you would like none of the time You accomplished less than you would like a little of the time You accomplished less than you would like some of the time You accomplished less than you would like most of the time You accomplished less than you would like all of the time
Pain	3.* During the past 4 weeks, how much bodily pain have you had?* None Very mild pain Mild pain Moderate pain Severe pain Very severe pain	*Pain in the past 4 weeks* You had no bodily pain You had very mild bodily pain You had mild bodily pain You had moderate bodily pain You had severe bodily pain You had very severe bodily pain
Vitality	4.* During the past 4 weeks, how much of the time did you feel worn out?* None of the time A little of the time Some of the time Most of the time All of the time	*Vitality in the past 4 weeks* You felt worn out none of the time You felt worn out a little of the time You felt worn out some of the time You felt worn out most of the time You felt worn out all of the time
Social functioning	5.* During the past 4 weeks, how much of the time has your physical health or emotional problems interfered with your social activities (like visiting with friends, relatives, etc.)?* None of the time A little of the time Some of the time Most of the time All of the time	*Social Functioning in the past 4 weeks* Your health limited your social activities none of the time Your health limited your social activities a little of the time Your health limited your social activities some of the time Your health limited your social activities most of the time Your health limited your social activities all of the time
Mental health	6.* During the past 4 weeks, how much of the time have you felt depressed or very nervous?* None of the time A little of the time Some of the time Most of the time All of the time	*Mental Health in the past 4 weeks* You felt depressed or very nervous none of the time You felt depressed or very nervous a little of the time You felt depressed or very nervous some of the time You felt depressed or very nervous most of the time You felt depressed or very nervous all of the time

While experts agree that evaluation of content validity of HRQoL patient-reported outcome (PRO) measures is advisable, preference-based measures such as the SF-6Dv2 have not been held to this standard. Evaluation of content validity includes evaluating the relevance (i.e., all items pertain to the construct of interest [generic HRQoL]), comprehensiveness (i.e., items cover all aspects of HRQoL), and comprehensibility (i.e., items are understood as intended) of PRO measures. These properties are evaluated through qualitative research methods during which individuals assess and provide direct feedback on each of these elements [18–22]. Although a review of the literature did not identify published studies of content validation of preference-based measures, the research team felt it was an important step in completing the development of the SF-6Dv2 HUS. Given the 8 domains measured by the SF-36 have been well established as those key to measuring HRQoL [23, 24], and recent evidence has shown the SF-6Dv2 to be conceptually equivalent to the SF-36 [25], this research focused on evaluating the comprehensibility of the SF-6Dv2 HUS.

### Objectives

This study had 2 objectives: (1) to evaluate the comprehensibility of the stand-alone SF-6Dv2 HUS (both Form A and Form B) by conducting individual cognitive debriefing interviews with adults in the general population of the United States (US); and (2) to learn which version of the stand-alone SF-6Dv2 HUS adults in the US prefer.

## Methods

### Study design

This was a qualitative, cross-sectional, non-interventional study consisting of one-on-one cognitive debriefing interviews. This approach to questionnaire evaluation is based in cognitive psychology and the Cognitive Aspects of Survey Methodology framework [26, 27]. Within this framework, questionnaire respondents are assumed to handle a number of cognitive tasks: (1) understanding the question(s) they are being asked; (2) retrieving their answer from memory; (3) internally evaluating their response; and (4) matching their response to the response options available in the survey. The cognitive debriefing interviews use a think-aloud approach that is designed to identify problems in comprehension that can be used to improve elements of the questionnaire.

The 75-min audio-recorded interviews were conducted by experienced qualitative researchers trained on the specific objectives of the study. All interviews were conducted by telephone or webcam; allowing for nationwide participation by a diverse geographic sample, while also alleviating health risks, given interviews took place during the COVID-19 pandemic. All study materials were approved by one central independent review board (IRB).^1^

### Study population

Eligible participants were age 18 and older, living in the US, and fluent in US English. Specific quotas were established to ensure a diverse and representative sample in: age (20 participants aged 18–49 and 20 aged 50+), sex (at least 5 males aged 18–49 and 5 males aged 50+, at least 5 females aged 18–49 and 5 females aged 50+), presence of chronic health conditions (at least 20 who answered yes), race/ethnicity (at least 10 identifying as non-white), and education (at least 10 participants with high school diploma or less). Participants were excluded from the study if they were unwilling or unable to participate in a single 75-min interview.

### Study procedures

All participants were recruited from the general population via a third-party recruitment vendor’s proprietary participant panel. All potential participants completed an online screening questionnaire to assess study inclusion criteria and standard demographic information. Participants who screened into the study were then directed to a second, brief questionnaire to collect further demographic information, and then scheduled for their interview. In total, 87 people were screened to participate. Recruitment was stopped when all quotas, including the total sample size of 40, was reached.

Interviews were conducted by one of two trained qualitative researchers with experience conducting cognitive debriefing interviews. At the beginning of each interview, the interviewer reviewed the consent statement in detail, answered any questions the participant might have, and asked for each participant’s verbal consent to participate. This was documented by each interviewer.

All interviews followed a standardized, semi-structured interview guide. Participants were randomly assigned one of the two forms of the stand-alone SF-6Dv2 HUS to debrief; half of the recruited sample (n = 20) debriefed Form A and the other half debriefed Form B. Interviewers used a think-aloud approach [28] to learn how well participants understood each aspect of the survey. During the think-aloud approach, participants were asked to read all parts of the survey—including title, instructions, items, and response choices—out loud and to say what they were thinking as they read the survey and answered the questions. If something was confusing to them, they were asked to describe what was confusing to them, and to articulate how they ultimately decided the meaning of the instruction, item, or response choice.

Following the think-aloud, participants answered a series of semi-structured follow-up questions about the various elements of the form they just completed, including instructions, recall period, items, and response choices. Responses to these questions, and spontaneous comments made during the think-aloud, were captured and later analyzed for evidence of each form’s comprehensibility. Lastly, participants were asked to review the alternate form of the stand-alone SF-6Dv2 HUS (i.e., whichever form they did not debrief earlier in the interview) and compare it to the one they had debriefed. They were then asked which form of the stand-alone SF-6Dv2 HUS they preferred, and why.

### Data coding and analysis

Data coding and analysis followed a 5-step process.

#### Step 1: quick code

Upon completion of each interview, the interviewer conducted a “quick code,” populating a Microsoft Excel spreadsheet with interview data solely from the interviewer’s field notes. Data included any notable issues that arose during the interview (e.g., confusing, or unclear items), suggested changes to either Form A or Form B, and overall preferences.

#### Step 2: cross-check transcripts

As completed transcripts were received, they were first reviewed for quality, and then cross-checked against the quick code spreadsheet to confirm all feedback had been accurately recorded during the quick coding process.

#### Step 3: code transcripts

Transcripts were then coded to identify additional information shared by the participants, including overall opinions on the stand-alone SF-6Dv2 HUS, and any other suggestions or insights. Coding was completed in NVivo software (QSR International Pty Ltd, 2018) and reviewed by the study PI. Coding reliability was determined through a consensus-based approach. The researchers independently coded the same first two transcripts and then met to review their coding and resolve any discrepancies through discussion. This meeting also served to allow for any initial adjustments to the codebook and code definitions. At this point, coding was consistent. The remaining transcripts were divided between the two coders and coded independently. The coders met throughout coding to ensure consistency and address any questions, and the study Principal Investigator reviewed all coding as an additional step to ensure coding reliability.

#### Step 4: analysis

All coded data were reviewed and analysed by the study team.

#### Step 5: review and consensus meetings

Determinations about potential modifications to Forms A and B were made through a consensus-based approach. The study team reviewed each of the issues identified or suggestions made by participants and noted the proportion of study participants who raised the issue/suggestion and the nature of their feedback (e.g., is the suggested edit crucial to improving comprehension or simply a matter of personal preference?). The research team evaluated each issue or suggestion—including whether it was raised spontaneously or as a response to a probe—and subsequently decided whether a modification was warranted.

All suggestions and supporting evidence for changes to either form were documented in an item tracking matrix [21, 29]. The matrix includes the original items from both forms, relevant comments suggesting a needed change, a decision on whether to change, how to change, and any new wording. The matrix also contains similar information on the instructions, recall period, and response choices.

## Results

### Participant demographics

A total of 40 individuals participated in this study. Most were white (n = 26, 65.0%), female (n = 23, 57.5%), had completed some form of post-high school education (n = 29, 72.5%), and had a chronic health condition (n = 29, 72.5%). Half of the sample was between the ages of 18–49, and the other half was age 50 or older. All participants were in the US including the Northeast (n = 12, 30.0%), West (n = 7, 17.5%), Midwest (n = 5, 12.5%), and South (n = 16, 40.0%). All participants were asked to rate their overall health; of those, fourteen (35.0%) rated their overall health as ‘very good’ or ‘excellent.’ Health satisfaction ratings were also collected and were wide-ranging across a 10-point scale, with an average of 5.8 out of 10 (min = 1, max = 9). (See Table 2).

**Table 2:** Demographic information

Demographics	N (%)	
*Sex*		
Female	23 (57.5%)	
Male	17 (42.5%)	
*Age*	Female	Male
18–29 years	1 (4%)	0 (0%)
30–39 years	9 (39%)	4 (24%)
40–49 years	2 (9%)	4 (24%)
50–59 years	9 (39%)	6 (35%)
60 + years	2 (9%)	3 (18%)
*Race/ethnicity*		
White	26 (65.0%)	
Black or African American	10 (25.0%)	
Asian	3 (7.5%)	
Hispanic/Latino/or of Spanish Origin	3 (7.5%)	
*Education*		
High school diploma or GED	11 (27.5%)	
Some college but no degree	5 (12.5%)	
Associate's degree or Technical Certificate	8 (20.0%)	
Bachelor's degree (BA, BS)	10 (25.0%)	
Graduate degree (MA, MS, PhD, MD, etc.)	6 (15.0%)	
*Has chronic health condition*		
Yes	29 (72.5%)	
No	11 (27.5%)	
*Region of residence*		
Northeast	12 (30.0%)	
West	7 (17.5%)	
Midwest	5 (12.5%)	
South	16 (40%)	
*Current work status*		
Retired	4 (10.0%)	
On disability or leave of absence	2 (5.0%)	
Temporarily furloughed	1 (2.5%)	
Unemployed, but looking for work	1 (2.5%)	
Employed full-time (40 h per week or more)	21 (52.5%)	
Employed part-time (less than 40 h per week)	8 (20.0%)	
Student (full- or part-time)	5 (12.5%)	
Stay-at-home parent or spouse	4 (10.0%)	
Other: Self employed	1 (2.5%)	

### Form A cognitive debriefing results

#### General assessment

All participants who debriefed Form A (n = 20), found it relevant, straightforward, and easy to understand. Participants were able to easily relate the questions to aspects of their daily lives and select an answer accordingly (see Table 3 for additional data).

**Table 3:** Form A and Form B cognitive debriefing results

Domain	Form A high-level summary—N (%)	Form A exemplary quotes	Form B high-level summary—N (%)	Form B exemplary quotes
General impressions	20/20 (100%): survey was clear and easy to understand	…I find the survey very interesting. I think they’re good questions to, um, kind of get a feel…a high-level feel for how someone’s feeling and dealing with things, you know, on a weekly basis. So you kind of get a…a high-level picture, you know, on my stress, on my anxiety, on my physical limitations, emotional limitations (ID27, male, 55 y/o)	18/20 (90%): survey was clear and easy to understand 2/20 (10%): survey was confusing or difficult to complete	I think it’s pretty comprehensive. It’s talking about your health in all terms of your personhood, um, with the exception of maybe spirituality. But it’s talking about your physical health. It’s talking about your emotional health. It’s talking about your state of mind. It’s talking about how energetic you feel and how your health is impacting your social life (ID20, female, 37 y/o)
Instructions and recall	20/20 (100%): instructions were clear and easy to understand 14/20 (70%): approved 4-week recall 4/20 (20%): recall was difficult due to COVID-19 2/20 (10%): recommended 2-week recall	…if you were to ask this in a non-global pandemic, um, it may have honestly been easier…I feel like all the days are kind of blending into each other right now [laughs] in life. Um, four weeks ago…so basically all of…well, the second half of October through right now. Um, uh—it was—it was fine. I—I think that if something really affected me, I would have recalled it. But I think for the last four weeks I’ve felt generally fine and so nothing really stuck out to me. I think four weeks is probably a fair, um, time frame for analysis (ID26, male, 39 y/o)	20/20 (100%): instructions were clear and easy to understand 18/20 (90%): approved 4-week recall 2/20 (10%): recommended 1–2-week recall	I liked that. Usually, people say six months, and you’re like, “Dude, six months. I have two kids.” Uhm, four, four weeks is, is very fresh, so it’s, I think it’s actually, uhm, a better period of time than, than knowing people say six months. Also, when, when you were not feeling well, six months ago is, you’ve had a time to change things. Four weeks is relevant (ID04, female, 37 y/o)
Physical Functioning	17/20 (85%): item was clear and easy to understand 3/20 (15%): item was confusing or difficult to answer	I don’t do heavy objects, I don’t run, I don’t play any, you know, strenuous sports. I do clean; I have moved tables in the last couple of weeks. You know, bathing and dressing is easy for me. So, those are really good examples of the differences between strenuous and moderate. Yes. They’re good (ID35, female, 55 y/o)	17/20 (85%): item was clear and easy to understand 3/20 (15%): item was confusing or difficult to answer	…it was easy. Yeah, they had descriptions for each one where it’s like, vigorous activity, then they had the moderate activities, so you kind of understand where they’re going with each thing. So…I understood it pretty clearly (ID24, female, 35 y/o)
Role Functioning	16/20 (80%): item was clear and easy to understand 4/20 (20%): item was confusing or difficult to answer	…certainly my physical health, um, does not conflict with my ability to accomplish my work tasks or my home tasks. But, uh, as I had mentioned briefly, my—my job is one where I tend to carry some people’s emotional baggage. Um, as they carry it, uh, to—to help them carry it, I guess. And over the last four weeks, there—there’s certainly been a lot of, uh, struggles, um, that I have had to carry for others, and which…I guess now that I think about it in a little more detail has—has certainly affected my, um, my work, which then I carried home with me. [laughs] Um, but, um, I wouldn’t say it affected my home regular activities, but it certainly did impact my work activities, yes. So if I were to focus solely on the emotional component of that question-It’s probably “some of the time.” (ID26, male, 39 y/o)	16/20 (80%): item was clear and easy to understand 4/20 (20%): item was confusing or difficult to answer	…like going to work and doing my job efficiently. Uh, I mean, even walking and driving, and, uh, going to the store, and cooking dinner and cleaning up, and you know, just, uh, every-day tasks (ID02, male, 38 y/o)
Pain	13/20 (65%): item was clear and easy to understand 7/20 (35%): item was confusing or difficult to answer	So, when you’re talking about pain, it needed to be description because I have RA, I have knee pain, I have asthma, I have stomach pain. It’s not descriptive and you have to tell me what kind of pain you’re talking about (ID 30, female, 56 y/o)	11/20 (55%): item was clear and easy to understand 9/20 (45%): item was confusing or difficult to answer	…the only difficulty I had is that it varies for me from day to day. Sometimes I have no pain, sometimes I have a lot of pain, sometimes it’s just a little. So, I try to kind of come up with a generalization over the last four weeks (ID08, female, 38 y/o)
Vitality	15/20 (75%): item was clear and easy to understand 5/20 (25%): item was confusing or difficult	…this was just me probably overthinking the question, but like is it health-related that I was worn out, or is it because I work nine hours a day in an office and then I came home and did such-and-such, such-and-such, or, you know what I mean? Why am I worn out? So ag—again, I’m probably overthinking it, and so that—that caused the only, um, you know, pause to think or maybe, you know, have to think a little harder before answering (ID34, female, 53 y/o)	17/20 (85%): item was clear and easy to understand 3/20 (15%): item was confusing or difficult	It sounded like, um, like, it says like, but, I guess like, I don’t really know. I have never heard that word before, but I, with the context of worn out it makes me think of, um, um, I thought worn out was, um, your level of like, um, tiredness, consciousness. I don’t know how to explain it. Like how you’re doing. Um, like how much juice or battery you still have, I guess. I don’t really know how to describe it…I think it might, I don’t even, I don’t, I’ve never heard the word before so I don’t know the definition. I was trying to use context (ID14, female, 19 y/o)
Social functioning	15/20 (75%): item was clear and easy to understand 5/20 (25%): the impacts of COVID-19 made this item difficult to answer	Well, when you’re talking about socializing in the, uh, in the age of COVID. So, I would have to just answer that question as if there was no COVID. Because, otherwise, we haven’t done that much, any socializing since, uhm, March, you know (ID33, male, 67 y/o)	15/20 (75%): item was clear and easy to understand 5/20 (25%): the impacts of COVID-19 made this item difficult to answer	That one was a little tricky because I was trying to think about it in the context of in normal life what would my health impact be on my social functioning? But I feel like we’re kind of in a…in a unique time period because I’m making decisions about my health and the health of other people around me due to the pandemic. Um, so ordinarily I would say my health doesn’t limit my social activities at all. But because of the situation that we find ourselves in and whether people are in quarantine or whether they’re not or whether they have health conditions that I’m trying to protect them from my germs or whatever, I feel like it does some of the time…I don’t know if you want the answer for my normal day-to-day life or if you want the COVID-19 social functioning (ID20, female, 37 y/o)
Mental Health	18/20 (90%): item was clear and easy to understand 2/20 (10%): item was difficult to answer due to outside circumstance	But it honestly, um, was it, it just seems like once the election was over and we kind of, kind of saw where it was going, it was a big sense of relief. And, so I wasn't feeling those feelings anymore. So that's why I was a little torn. But I did pick a little of the time because it was leading up to the election and it does say, and it does say the past four, four weeks (ID35, female, 55 y/o)	12/20 (60%): item was clear and easy to understand 8/20 (40%): item was difficult to answer due to variability of mental health over 4 weeks	That was actually pretty difficult. Um, I’m diagnosed, I’m diagnosed bipolar, so trying to calculate my episodes or like the map of my mood is really, it’s difficult to remember and just to keep track of in general. So, answering that, especially for four weeks, was quite difficult…I was trying to think like which, like which time, like when was I feeling like nervous or depressed. So, because I, sometimes I’m not and it’s hard to like map out like exactly how long that went for, or how long that was (ID19, female 33 y/o)

#### Instructions and recall

All participants found the instructions for Form A clear and easy to understand. One participant initially missed the instructions but was able to complete the survey with no issues.

Fourteen participants found it easy to recall how they were feeling over the past 4 weeks and to answer each question within that timeframe. Of the 6 who did not, 4 recommended shortening the recall period to 2 weeks and 2 participants felt it was difficult to recall the past 4 weeks due to monotony of the previous months (related to the COVID-19 pandemic) but did not provide an alternative recall period.

#### Individual items

##### Physical functioning

Overall, participants found the physical functioning question easy to answer (n = 17). Of those who found it difficult (n = 3), 1 participant felt it was unclear whether the response choices were mutually exclusive (i.e., if they are limited a little in moderate activities, does that mean they cannot do vigorous activities?); another did not engage in vigorous activities and could not answer whether they were limited; and another was unsure how to answer the question because their multiple chronic health conditions limited them in different ways.

##### Role functioning

Most participants found the role functioning question easy to answer (n = 16). Of the 4 participants who had difficulty answering the question: 1 struggled with recalling regular daily activities over the past 4 weeks, another felt the question was too wordy and suggested changing the wording to “felt or were less productive,” 1 felt their answer would differ depending on whether they focused on work or activities outside of work, and another suggested splitting the question into 2 separate items (1 for physical health and 1 for emotional problems). However, upon further questioning, all participants were able to understand and interpret the question accurately.

##### Bodily pain

Just over half of participants found this question easy to answer (n = 13). The other 7 found it difficult for a variety of reasons. Three struggled to recall their pain over the past 4 weeks—with 2 noting their pain fluctuated requiring them to come up with an average pain level so they could answer the question. While able select a response for this item, 2 participants found it difficult to do so quickly, as they felt the question and response choices were too subjective (i.e., definitions of pain will be different and so answers cannot be accurately compared). Two participants were unsure whether the question was asking about acute or chronic pain and felt their answers would differ depending on the type of pain.

##### Vitality

Most participants (n = 19) found the vitality question easy to answer, although 4 took a longer time to select an answer as compared to previous items. The 1 participant who had difficulty answering struggled with recalling times when they felt worn out over the past 4 weeks. Additionally, 2 participants felt the phrase “worn out” was too vague and should specify whether it includes emotional problems or just physical health, however upon further probing each person considered both physical health and emotional problems when answering the question.

##### Social functioning

Fifteen participants found the social functioning question easy to answer. The 5 participants who found it difficult to answer referred to the COVID-19 social distancing restrictions in place at the time of the interviews. Because social activities were restricted due to local ordinances, these participants experienced interference with social activities in the 4 weeks prior to the interviews. Although the interference was not due to physical health or emotional problems, it made it difficult for them to answer this item, nonetheless.

##### Mental health

Overall, participants found the mental health question easy to answer (n = 18). Of those who found it difficult (n = 2), 1 participant felt it was hard to admit, and be vulnerable enough to answer the question, while the other felt the current state of the world (e.g., ongoing COVID-19 pandemic) made it difficult to answer the question.

### Form B cognitive debriefing results

#### General assessment

Overall, most participants who debriefed Form B (n = 18) were able to easily relate questions to aspects of their daily lives and answer accordingly, and thought it was straightforward and easy to understand, with only 2 participants finding the form confusing or difficult to answer. Of these 2 participants, 1 struggled with whether to consider their health pre-COVID-19, or if they should answer in the present day, while the other was unsure what the survey was asking overall and therefore had a difficult time selecting statements that described them (see Table 3 for additional data).

#### Instructions and recall

All participants found the instructions for Form B clear and easy to understand. Most participants (n = 18) found it easy to recall how they were feeling over the past 4 weeks and had no difficulty answering each question within that timeframe. Of the 2 who did report issues, 1 recommended shortening the recall period to 2 weeks, while the other suggested it would be easier to remember the past 1–2 weeks, rather than the past 4.

#### Individual items

##### Physical functioning

Overall, the physical functioning question was found to be clear and easy to answer (n = 17). Three participants (out of 20) found it difficult to answer, primarily due to general confusion over which statement best described their health and the circumstances limiting their physical functioning.

##### Role functioning

Role functioning was perceived as easy to answer (n = 17). Participants interpreted “ability to work and do regular daily activities” to mean their general responsibilities as an employee, parent, or member of society, including going to work and completing household chores. Participants who found this item difficult to answer (n = 3) found the double negative statement to be confusing (i.e., you accomplished less than you would like none of the time; n = 1) and had different answers for physical and emotional health and would have preferred to answer each separately (n = 2).

##### Bodily pain

Similar to Form A, just over half of the participants (n = 11) found this item easy to answer. The 9 participants who did not reported this item was difficult to interpret and found it challenging to distinguish between the response choices mild and moderate (given the response choice of very mild), and severe and very severe. Participants also had difficulty averaging their pain over 4 weeks given daily fluctuations. One participant was unsure if the item is referring to chronic or acute pain, which made selecting a statement to describe their pain difficult.

##### Vitality

Overall, the item on vitality was easy for participants to answer (n = 17), although 3 reported finding it difficult to select a statement to describe themselves. These participants were confused over what “worn out” was referring to (e.g., does being tired at the end of a busy day qualify?). Participants also questioned the meaning of the heading (“Vitality”) and whether the concept is easily recognizable; ultimately, it was interpreted to mean being worn out mentally, worn out physically, or both.

##### Social functioning

Similar to the results for Form A, participants found the social functioning item in Form B easy to complete (n = 15), however the ongoing COVID-19 pandemic added difficulty for some individuals (n = 5). Participants who indicated this was difficult to answer noted that all social activities were limited, regardless of their health, making it challenging to decide which statement to select. Participants who found this item easy to complete also brought up COVID-19-related social restrictions, however it did not impede their ability to select a statement, or their understanding of the item.

##### Mental health

While 12 participants had no difficulty with the mental health item in Form B, 8 participants found it difficult to select a statement to describe their mental health. Four described their feelings of depression or anxiety as variable and found it difficult to select one statement to best describe them over the past 4 weeks. Some participants (n = 3) also had difficulty selecting a statement if they experienced only depression or only anxiety. The double-barreled nature of the item wording made it difficult to choose the most appropriate statement. Similarly, 2 participants found the word “depressed” to be triggering, articulating there is a difference between being depressed and feeling depressed and it isn’t clear which the item is referring to. Finally, 1 participant found this item difficult to answer in an interview setting with a stranger, while another wasn’t sure how to answer given how the COVID-19 pandemic has influenced all aspects of life.

### Comparison of Form A and Form B

**Fig. 1:**
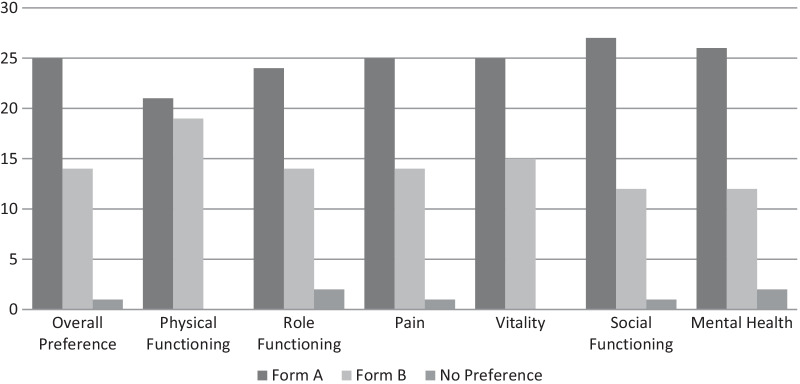
Form comparison

There was a general tendency to prefer the last form the respondents had seen. Of the 20 participants who debriefed Form A, 12 preferred Form B *after* reviewing Form B, while only 7 preferred Form A. One participant had no preference. Of the 20 who debriefed Form B, only 2 preferred Form B *after* reviewing Form A, while 18 preferred Form A. Taking this recency effect into account, more participants preferred Form A above Form B (See Fig. 1 and Table 4).

**Table 4:** Form comparison—participant quotes

Category	Preference Form A	Preference Form B
Overall comparison	It just seems more clear in the way they, like I do this in some of my emails, uh, you know like underline and bold and things like that. It just makes it, and then they’re more descriptive with what they mean by different things (ID29)	I think I like, uh, the—the first one, the [Form B] rather than [Form A]. I just like the way it’s easier to see, and I like the way that it’s laid out, though of course, that’s a personal preference. So for me, I like the way that this one is laid out better (ID07)
Physical functioning	Uh, the first option, the “does your health now limit you in physical activities?” I don’t know if it’s the way that it’s worded or the way…like the options seem more succinct and clearer, “not limited at all, limited a little,” and then it goes down to “limited a lot in bathing and dressing.” I don’t know if it is different, but it feels different than the first survey. Oh no, it’s not that different. There’s less words (ID20)	… you’re getting all those parameters with the question rather than having to jump between the answer and back to the question. So, your question is included in the choice. It’s a little easier for me to follow. Maybe because of the way I look at things, uh, but that’s—the organization is easier to follow and more—more cogent to me, in 1.4 [Form B] (ID13)
Role functioning	Um, the last version was kind of like in general, you know. It’s kind of broad. This is a little more specific, like asking would you…have…do you feel like you’ve accomplished less than you would like at work or during other regular activities (ID40)	Yes, I would. I—I just like the way it looks better. But, you know, that you—you ask me and then you ask the next person and they’re going to tell you totally different. It’s just a personality thing I think, or a—or a, um, because an artist—I may look at something different than somebody who is a mathematician, you know. [laughs] So I don’t know how that’s going to help you. But anyway, that’s for me…my personal preference is [Form B] (ID07)
Pain	I like it scaled down like this because it’s easy to read, “past four weeks, bodily pain.” (ID16)	…I think like the emphasis is different by putting it in that sentence over and over where you could like say, “Oh, that’s me,” versus just thinking… I don’t know, it just felt like it was more organized than the how many, you know, how much body pain have you had, and then it was just like listed there (ID19)
Vitality	…it definitely kind of gets to the point of in the past month how often did you feel tired, or exhausted or worn out, and I think – you know, again, the format of it I like, and also the fact that, you know, you – it, it really flat out just asks you the question, uh, there's no second guessing. It's easy to follow (ID02)	Definitely this one, [Form B]…Because it has, because it has the word vitality in it. Uh, um, that’s just me personally (ID01)
Social functioning	I don’t think so. Like question five I feel like in [Form A] was easier for me to answer than in [Form B], I think because of the example. Because when I was answering that on [Form B], I wasn’t thinking like social activities involving like my family. I was, I don’t know why. It just didn’t even occur to me. I thought we were talking about something completely like separate from like family gatherings and things like that. Um, so I feel like in, in [Form A], that question I could answer more clearly (ID08)	Uh, I think, I think it’s the same reason, and I think it’s easier to read through almost, to where it’s like, it’s not, you don’t have to ask, it’s not like the question and then all the answers. It’s the same question so you can read through it quick, and then the answer is down (ID11)
Mental health	I prefer [Form A] because it doesn’t have the word “mental health” in it. And I think that sometimes as a society, we have issues with mental health. And to put that—I think it’s usually a negative connotation with the word “mental health.” Um, it’s seen as a problem rather than just a part of who we are. Um, so when I think of mental health, it’s generally in a negative sense. So to have that in question [Form B], I’m already thinking about it negatively rather than thinking about it neutrally (ID20)	Um, I like it, actually, on the [Form B]. It looks more professional to me. Mental health in the past four weeks, other than, you know, it was very kind of drawn out on the [Form A]. During the past four weeks, how much of the time have you felt depressed or nervous—or very nervous? This one is just saying, mental health in the past four weeks. And so, and then, you guys are asking the question. You felt very depressed or very nervous, none of the time? You felt depressed or very nervous, all of the time? So, to me, it made more sense, looked more professional, on [Form B] (ID05)

Overall, participants found the items in Form A clearer and easier to answer. When comparing Forms A and B, more participants preferred answering questions (Form A) over choosing from a set of statements (Form B). The numbered questions and underlining of key words in Form A fostered quick and easy comprehension and completion of the survey. Participants also felt it looked more professional and was more in line with what they were used to seeing. While participants found some of the titles in Form B to be helpful, overall, they preferred the questions in Form A. Overall preference mostly aligned with participant preferences for individual items within the two forms. Individuals whose overall preference was Form A, also tended to prefer the question/answer items in Form A over the corresponding statement items in Form B and vice-versa. However, this was not always the case.

Despite an overall preference for Form A, almost half of participants (n = 19) preferred the physical functioning question in Form B, finding it clearer and easier to answer. They found it helpful to have the descriptions of vigorous and moderate activity in the response choices (n = 11), and they found the wording easier to understand (n = 9). Eight participants found the response choices in Form A to be challenging when comparing them to Form B.

## Discussion

This qualitative study was designed to elicit feedback from US adults on the overall comprehensibility of 2 different Forms (A and B) of the stand-alone SF-6Dv2 HUS Form and on which form they prefer. The study provided strong evidence that both forms of the stand-alone SF-6Dv2 HUS were understandable and easy to complete. There were no difficulties with instructions or recall period on either form; however, participants expressed preference for Form A, finding it easier to complete. The only exception was the physical functioning item, for which participants preferred the format of Form B. In Form B, the definitions of vigorous and moderate activity are included in the response choices, which participants preferred over the format of Form A.

Given the overall feedback, we decided to move forward with Form A, but with revisions to the physical functioning item to make it more like Form B, ensuring it is easier to understand (see Table 5). Specifically, the definitions of vigorous and moderate activity were moved from the question stem to the response choices, as participants found having the definitions in the response choices made it easier to select an answer. Although both forms had items participants found difficult to answer (on average, 4 participants (18%) had difficulty with Form A and 5 participants (26%) had difficulty with Form B), none of these difficulties prevented them from completing the survey, nor did they warrant further changes to the items or response choices. One respondent raised the issue whether the categories describing levels of physical function were mutually exclusive. This issue was analyzed in detail during the development of the SF-6Dv2 [17] as well as in previous analyses of these physical function items [30]. These analyses strongly support that the health levels of the PF item forms a clear hierarchy assessing one overall construct of physical function. We believe that the revised descriptions of the levels of physical function clarifies this hierarchy.

**Table 5:** Modification to physical functioning item

Category	Original Item: Form A	Modified Item: SF-6Dv2 Health Utility Survey
Physical functioning	7.* Does your health now limit you in your physical activities*, for example vigorous activities (such as running, lifting heavy objects, participating in strenuous sports), moderate activities (such as moving a table, pushing a vacuum cleaner, bowling or playing golf), or bathing and dressing? Not limited at all in vigorous activities Limited a little in vigorous activities Limited a little in moderate activities Limited a lot in moderate activities Limited a lot in bathing and dressing	1.* Does your health now limit you in your physical activities?* No, not limited at all in vigorous activities, such as running, lifting heavy objects, participating in strenuous sports Yes, limited a little in vigorous activities Yes, limited a little in moderate activities, such as moving a table, pushing a vacuum cleaner, bowling or playing golf Yes, limited a lot in moderate activities Yes, limited a lot in bathing and dressing

Of particular interest during cognitive debriefing was the participant feedback on the bodily pain items. While no participants asked for clarification on the bodily pain items when completing either form, some participants reported issues with the items during think-aloud. Of those who debriefed Form A, two found the response choices “too subjective”; two noted challenges with recalling pain over the last 4 weeks; and two struggled to determine if the item was asking about acute or chronic pain. Since the chosen version of the BP item is identical to the first item of the SF-36 (and SF36v2), these results should be considered in light of the body of studies on the validity of this bodily pain item. Psychometric analyses have supported that the response choices of the BP item define separate levels of pain [31]. The issue of length of recall has been examined by comparing different version of the BP item with an average of momentary assessments covering the same time frame [32]. Strong correlations were found between average momentary assessment and all lengths of recall. Highest correlation was seen for 1 day recall, followed by 3 days, 4 weeks and 1 week recall. Four-week recall had higher correlation with momentary assessment than 7-day recall [32]. On a pragmatic level, the optimal recall will depend on the population and intended use of the instrument. For conditions where pain is episodic rather than constant, a too short recall period may lead to high variability in the assessment of pain, unless the instrument is administrated very frequently. For these reasons, we decided to keep the current version of the bodily pain item in the standard version of the SF-6Dv2 HUS, but to also suggest an additional version of the SF-6Dv2 HUS, using a 1-week recall for all the items where a recall is specified.

This study had several limitations. The sample was based in the United States and the study data collection took place in October and November 2020. Participants indicated that their answers to the social functioning and mental health items were influenced by external factors including the ongoing COVID-19 pandemic, surging cases of COVID-19 in some regions of the US, COVID-19-related social distancing policies and restrictions, and stress regarding the contentiousness of 2020 US presidential election. Although these factors influenced the participants’ answers, they had no bearing on their ability to understand the items and select responses. Additionally, due to COVID-19 travel and social-distancing restrictions, all interviews were conducted by phone or webcam; it is typically preferable to conduct as least some interviews in person.

This study had unique strengths, including the characteristics of participants. Recruitment quotas ensured a representative sample, including a wide age range of participants of both sexes, participants with the equivalent of a high school education or less (27.5%), and participants with chronic health conditions (72.5%), including diabetes, hypertension, hyperlipidemia, chronic pain, HIV, arthritis, depression, and anxiety. The decision to randomize which form of the stand-alone SF-6Dv2 HUS participants debriefed was an additional strength. Randomizing the order controlled for the possibility of recency effects impacting the results. The number of interviews conducted (n = 40) is a further strength, supporting the comprehensibility of the stand-alone SF-6Dv2 HUS for use with a general population of adults. While 7–10 cognitive debriefing interviews can be sufficient to determine comprehensibility of an instrument, testing the SF-6Dv2 HUS in 40 interviews ensures it can be used with a diverse population [29]. While identified above as a limitation, it is also a strength that participants were able to consider health-related impacts on their social function versus pandemic-induced impacts. This differentiation confirmed their understanding of the items and the concepts being measured.

Finally, testing the stand-alone SF-6Dv2 HUS with participants as part of the development process was an additional strength of this study. There is scant published literature on health utility survey measures documenting testing with participants during development, yet this testing is an important step to confirm the comprehensibility of the measure [18–21]. Furthermore, this testing ensures all aspects of the survey (including instructions, questions, and response choices) are understandable and easy for patients to complete.

The development of the stand-alone SF-6Dv2 HUS is a key addition to the field of HRQoL. Health utility measures are widely used by health regulatory agencies, and systems that review approval for payment of medication or conduct comparative effectiveness research. A brief, easy to administer, stand-alone SF-6Dv2 can be more easily implemented and interpreted across patient groups and disease areas than its predecessor, aligning it more closely with the usefulness of the EQ-5D and HUI. Current research is evaluating the psychometric properties of the stand-alone SF-6Dv2 HUS to confirm this in the general population, and further research should be done to confirm it within specific disease populations.

## Conclusions

In conclusion, the stand-alone SF-6Dv2 HUS is an understandable and easy to use assessment of HRQoL intended for use with adults. Use of the stand-alone SF-6Dv2 HUS can contribute to the comprehensive assessment of a patient’s health status and administrators can feel confident it is measuring the intended concepts with fidelity while minimizing patient burden.

## Data Availability

Specific data points can be made available upon reasonable request.

## Abbreviations

FDA: Food and Drug Administration
GPBM: Generic preference-based measure of health
HRQoL: Health-Related Quality of Life
IRB: Independent Review Board
MCS: Mental component summary
PCS: Physical component summary
QALY: Quality-adjusted life-years
QM: QualityMetric Incorporated, LLC
US: United States

## Footnotes

1 New England IRB (NEIRB) Study #1293768; Given that this study posed minimal risk for study participants, NEIRB approved a waiver of signed consent.
